# Parametric analysis of occupant ankle and tibia injuries in frontal impact

**DOI:** 10.1371/journal.pone.0184521

**Published:** 2017-09-14

**Authors:** Fuhao Mo, Xiaoqing Jiang, Shuyong Duan, Zhi Xiao, Sen Xiao, Wei Shi

**Affiliations:** 1 The State Key Laboratory of Advanced Design & Manufacturing for Vehicle Body, Hunan University, Changsha, China; 2 CRRC Zhuzhou Institute CO., LTD, Zhuzhou, China; 3 School of Mechanical Engineering, Hebei University of Technology, Tianjin, China; Beihang University, CHINA

## Abstract

**Objective:**

Non-fatal tibia and ankle injuries without proper protection from the restraint system has gotten wide attention from researchers. This study aimed to investigate occupant tibia and ankle injuries under realistic frontal impact environment that is rarely considered in previous experimental and simulant studies.

**Methods:**

An integrated occupant-vehicle model was established by coupling an isolated car cab model and a hybrid occupant model with a biofidelic pelvis-lower limb model, while its loading conditions were extracted from the realistic full-frontal impact test. A parametric study was implemented concerning instrument panel (IP) design and pedal intrusion/rotation parameters.

**Results:**

The significant influences of the IP angle, pedal intrusion and pedal rotation on tibia axial force, tibia bending moment and ankle dorsiflexion angle are noted. By coupling their effects, a new evaluation index named CAIEI (Combined Ankle Injury Evaluation Index) is established to evaluate ankle injury (including tibia fractures in ankle region) risk and severity in robustness.

**Conclusions:**

Overall results and analysis indicate that ankle dorsiflexion angle should be considered when judging the injury in lower limb under frontal impact. Meanwhile, the current index with coupling effects of tibia axial force, bending moment and ankle dorsiflexion angle is in a good correlation with the simulation injury outcomes.

## 1. Introduction

Although the number of fatal injury cases has been dramatically reduced in recent decades, the lower limb injury severity is still at a high level. Annual incident analysis report [1] showed that lower limb injuries accounted for 32% of AIS 2+ injuries in 2010. Moreover, seatbelts and safety airbags cannot effectively reduce the risk of lower limb injuries, which was different from its effective protection for other fatal injuries related to head or chest [2–3]. China Automotive Technology and Research Centre (CATARC) found that the lower limb injury score of 100% full frontal impact for Chinese brand cars was only 49.1% in the China-New Car Assessment Program (C-NCAP), compared to 65.6% for Japanese brand cars and 65.1% for European/American [4–5]. Rudd [6] also indicated that occupants accounted for 82.5% of lower limb injuries according to accidental statistical analysis in the United States from 1994 to 2007. Otte [7] showed that 42% of lower limb injuries occurred in foot/ankle regions in frontal collisions. Chong [8] found that significantly higher percentages of 'open' fractures were recorded in the below knee area among all lower extremities injuries. However, occupant lower limb kinematics in frontal crashes is complicated and the mechanisms related to ankle and tibia are still controversial regarding to real impact environments [6, 9].

Occupant ankle and tibia injury mechanisms and tolerances have been widely discussed in previous decades. This kind of injuries are mostly due to the direct impact from the pedal or vehicle interiors’ invasion. Morgan [10] found that 43% of occupant ankle injuries were caused by pedal contact force. Meanwhile, 12% of ankle injuries were caused by the contact force from the instrument panel (IP) or the dash panel. Thomas [11] indicated that the tibia injury risk would increase by 54% when the size of pedal intrusion was bigger than 200 mm. Besides the intrusion in backward and upward direction, ankle rotation is also a potential factor leading to serious ankle injuries [12–13]. As rarely considered in previous studies, the contribution of the whole occupant lower limb kinematics to ankle and tibia injuries in the frontal crashes. Ankle and tibia injury mechanisms and tolerances related to realistic impact conditions should be further studied.

Many studies have been conducted to determine ankle and tibia injury thresholds by considering ankle rotation angle, force or moment. Portier [14] indicated that the average ankle torque for ankle ligament injuries is 60 Nm. Shin [15] showed that the most vulnerable position for occupant ankle is in internally rotated 15°. Begeman [16] found that tibia fractures happen with average axial force reaches 7.59 kN. Yoganandan [17] studied derived axial loading-induced injury risk curves based on survival analysis using peak force. Rudd [18] indicated that the coupling effect of an ankle joint moment of 59 Nm as well as the ankle dorsiflexion angle of 36° represented a 25% injury risk of ankle injury for a 50th percentile male. Most of previous studies are based on isolated segment tests with simplified loading conditions. The whole human body kinematics related to realistic car crash conditions and its influences on ankle injuries is less considered. Furthermore, the coupling effects of different injury indexes such as ankle rotation, bending moment and compression force are also not discussed thoroughly.

Hence, this study integrated a biofidelic lower limb model and a realistic car cab model with seatbelt and airbag restraint system to analyse occupant ankle and tibia injuries in frontal impact. The whole lower limb kinematics influenced by the safety system and vehicle structure invasions were simulated. An orthogonal simulation test considering automotive interior design and invasive parameters of the cabin were designed to fully investigate their influences on ankle and tibia injuries. At last, a new injury evaluation index was proposed for ankle injuries considering coupling effects of ankle dorsiflexion angle, tibia bending moment and axial force.

## 2. Materials and method

### 2.1 Integrated occupant-vehicle FE model

A whole occupant-vehicle model (S1 Fig) was established by integrating three major subsystems including an isolated middle size car cab model (Chinese brand), a hybrid occupant model combing a dummy model and a biofidelic pelvis-lower limb model (S1 and S2 Tables) at the pelvis central point as well as a car restraint system consisting of a three-point seatbelt and a frontal safety airbag.

All boundary conditions were based on the experimental results of the C-NCAP full frontal impact at the speed of 50 km/h. The effect on the occupant by this impact was simulated by imposing an impact acceleration on the hybrid occupant model. The whole model was then validated by comparing with test results including occupant kinematics, human body acceleration and belt force [19]. The evaluation of the integrated occupant-vehicle FE model in whole occupant kinematics was shown in S2 Fig.

### 2.2 Selection of the parameters

**Fig 1:**
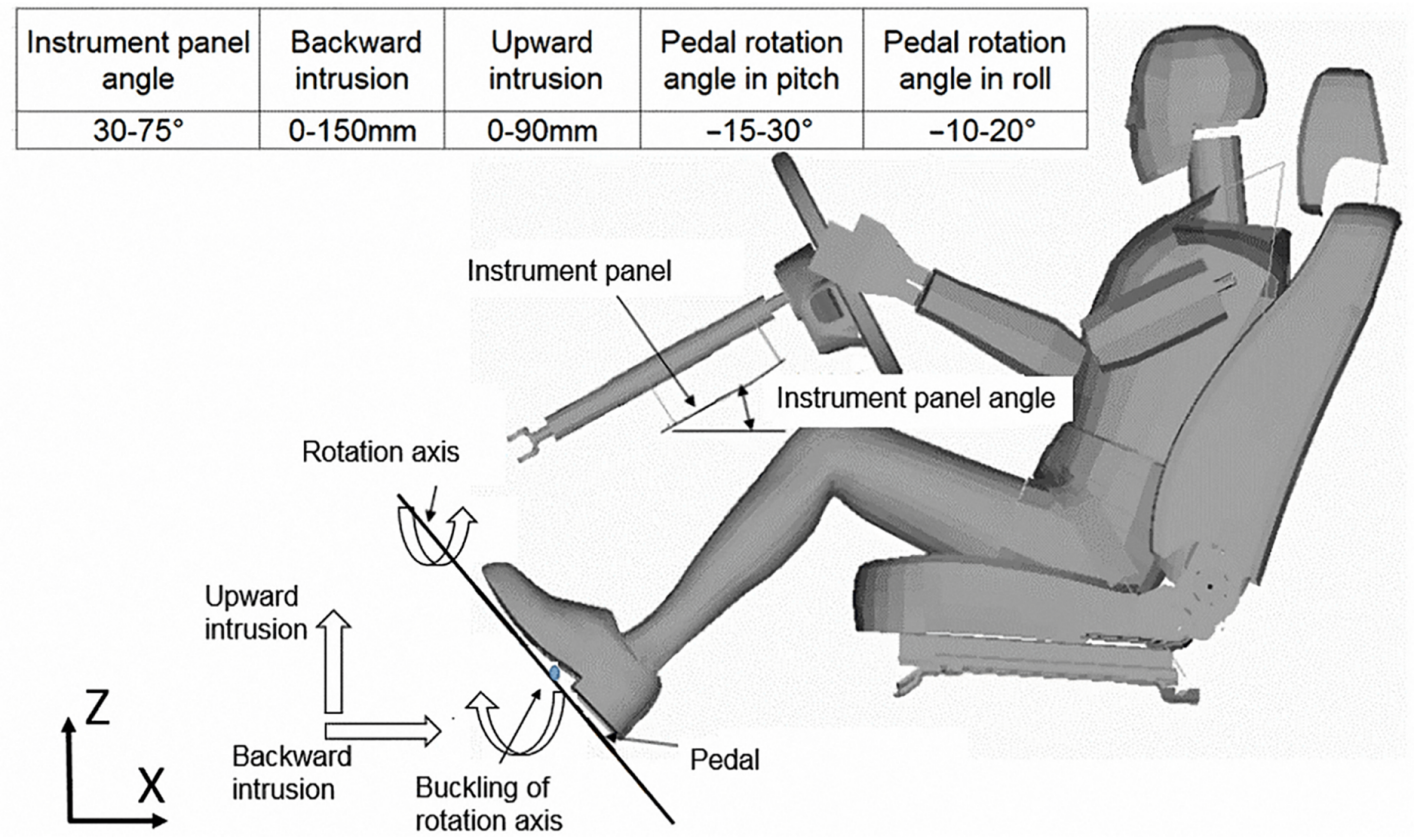
Parameter setting of the simulation matrix on tibia and ankle injuries.

Concerning ankle and tibia injury mechanisms, five parameters were selected for the parametric study such as IP angle, pedal intrusions in upward and backward directions, pedal rotations in pitch and roll (Fig 1).

The IP angle was set 30°-75° according to the design. Based on the NCAP test analysis [20], the intrusion limits in the backward and upward direction were selected at 150 mm and 90 mm, respectively. Pedal rotation angle in pitch was decided from -15° to 30° and the forward direction was negative. Meanwhile, the pedal rotation angle in roll was chosen from -10° to 20°and the outward direction was negative.

### 2.3 Design of the parametric study

Orthogonal test method was used to study the sensitivity of five parameters on ankle and tibia injuries. A L16 (4^5^) orthogonal experimental table was employed, as shown in Table 1. A, B, C, D and E represent the factors of IP angle, pedal backward intrusion, pedal upward intrusion, pedal rotation angle in pitch and pedal rotation angle in roll, respectively. Each fact has four levels. Fractures and several physical indexes were recorded, including tibia axial force, tibia bending moment and ankle dorsiflexion angle. Totally,16 simulations were conducted using LS-DYNA codes.

**Table 1:** Simulation matrix of the parametric study.

Simulation	A (°)	B (mm)	C (mm)	D (°)	E (°)
*1*	30	0	0	-15	-10
*2*	30	50	30	0	0
*3*	30	100	60	15	10
*4*	30	150	90	30	20
*5*	45	0	30	15	20
*6*	45	50	0	30	10
*7*	45	100	90	-15	0
*8*	45	150	60	0	-10
*9*	60	0	60	30	0
*10*	60	50	90	15	-10
*11*	60	100	0	0	20
*12*	60	150	30	-15	10
*13*	75	0	90	0	10
*14*	75	50	60	-15	20
*15*	75	100	30	30	-10
*16*	75	150	0	15	0

## 3. Results

**Fig 2:**
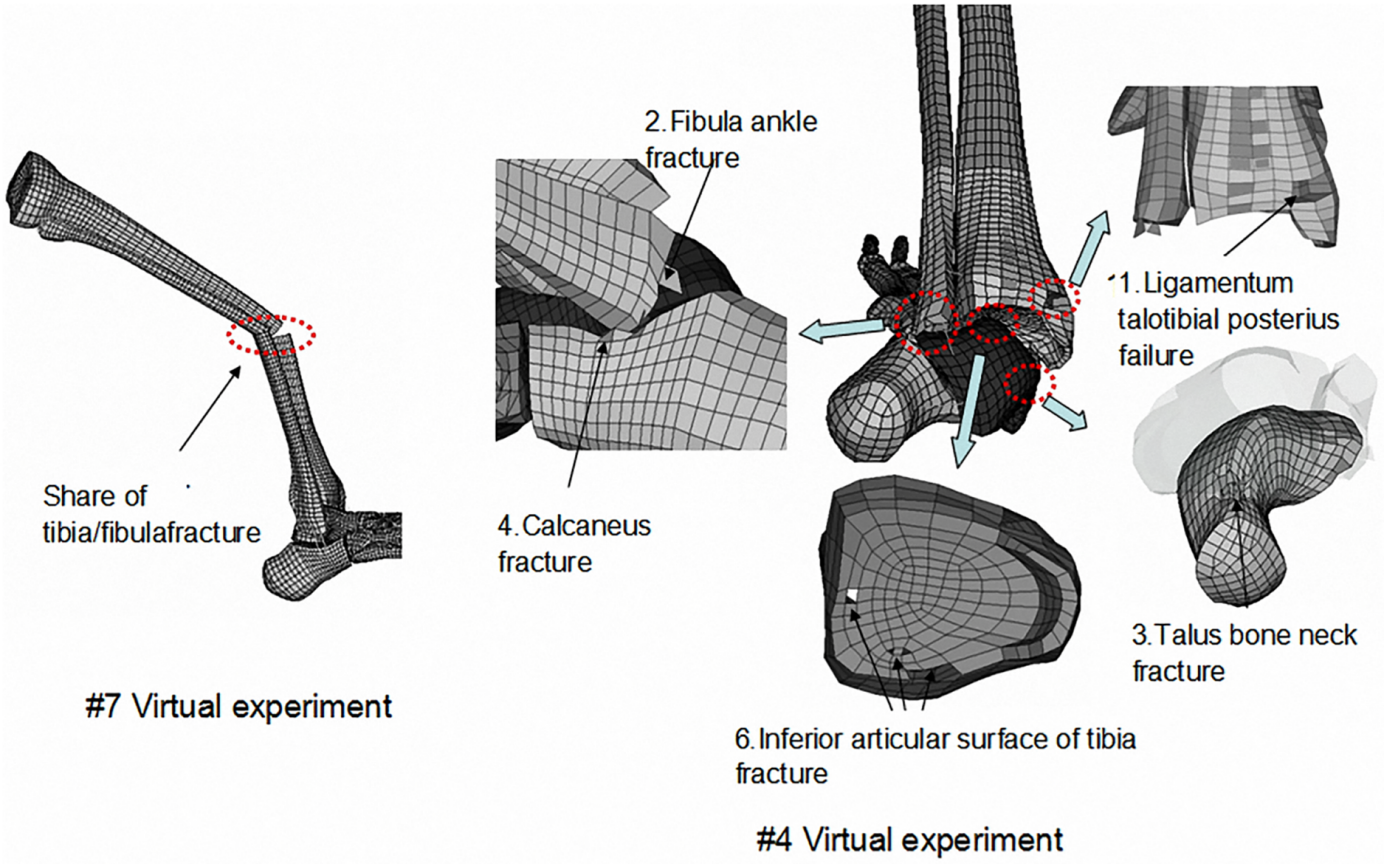
Examples of various injury types in the parametric study.

There was no fracture found in 5 cases of all the 16 simulations. Some injuries were found in ankle and tibia regions in other 11 cases including talus neck fracture, fibula condyle fracture, calcaneotibial ligament failure, tibial posterior cruciate ligament rupture, as shown in Fig 2.

Some examples of injury indexes like tibia axial force, bending moment and ankle dorsiflexion angle in each simulation were listed in Table 2. Multiple injuries were found in most cases. Furthermore, mostly noticed injury occurrence was the failure of the tibia posterior cruciate ligament and most fracture cases were distal tibia fractures located in the ankle region.

**Table 2:** Left ankle/tibia injury outcomes (maximum) in the parametric study.

Simulation	Ankle dorsiflexion angle (°)	Tibia axial force (kN)	Tibia bending moment (Nm)	Injury*	RTI	CAIEI
*1*	7	0.95	63	none	0.34	0.27
*2*	38	2.95	154.6	1	0.89	0.97
*3*	49	2.7	141	1 2 3 4 6	0.81	1.09
*4*	72	2.15	124	1 2 3 4 5 6	0.70	1.35
*5*	40	2.02	94.8	1 6	0.56	0.84
*6*	50	1.98	122.3	1 2 3 5 6	0.67	1.03
*7*	28	4.1	142	7 8	0.93	0.86
*8*	27	2.88	128	3 6	0.77	0.76
*9*	48	2.53	99.2	1 2 3 4 6	0.62	0.98
*10*	45	4	139.5	1 3 6	0.91	1.08
*11*	34	1.91	114	none	0.63	0.79
*12*	22	1.48	100	none	0.54	0.57
*13*	26	2.5	122	none	0.72	0.72
*14*	8	1.69	152.6	none	0.78	0.50
*15*	52	2.84	116.6	1 2 3 4 6	0.72	1.08
*16*	46	2.59	125.4	1 2 3 4 6	0.74	1.01

*Injury types: 1. Ligamentum talotibial posterius rupture; 2. Fibula ankle fracture; 3. Talus bone fracture; 4. Calcaneus ligament rupture; 5. Calcaneus fracture; 6. Inferior articular surface of tibia fracture; 7. Tibia fracture; 8. Fibula fracture.

The average value of the outcomes was applied to represent the value of one input factor when analysing the influences of the study parameters on axial force, bending moment and ankle dorsiflexion angle. For example, the average value of tibia force in simulation 4, 7, 10 and 13 was 3.19 kN, which was used to represent the pedal upward intrusion at 90 mm.

### 3.1 Tibia axial force and bending moment

**Fig 3:**
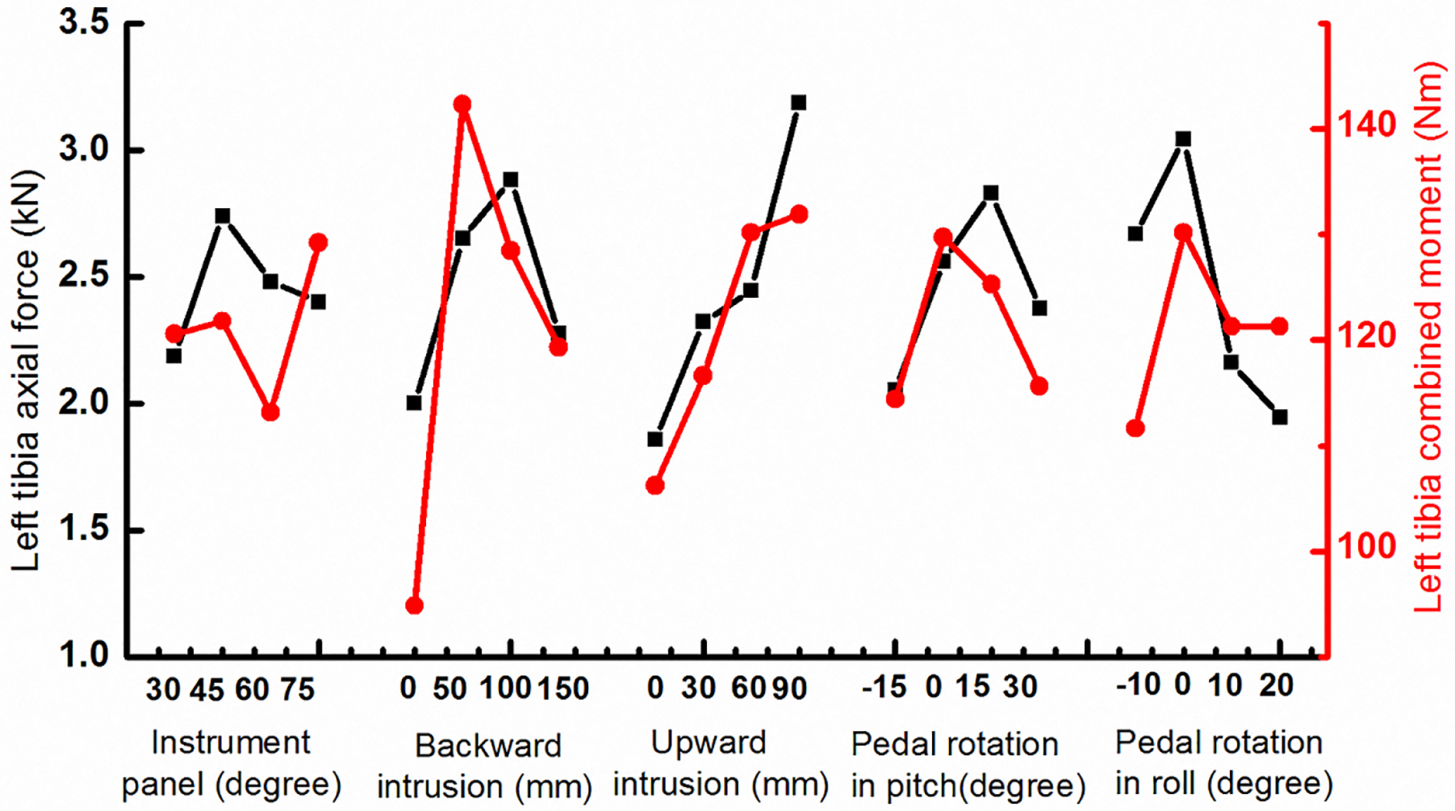
The influences of the study parameters on left tibia axial force and bending moment.

The influences of the study parameters on tibia axial force were shown in Fig 3. Compared with the extreme deviation of five groups, the upward intrusion presented most important influences on tibia axial force while its extreme deviation reached by 1.71 kN. The tibia axial force increased sharply with the increase of the upward intrusion, from 1.86 kN at 0 mm intrusion to 3.18 kN at 90 mm intrusion. Such a trend did not happen when concerning the influence of the pedal backward intrusion or other parameters. The maximum tibia axial force appeared while the IP angle was at 45°, the pedal backward intrusion at 100 mm, the upward intrusion at 90 mm, the pedal rotation angle in pitch at 15° and the pedal rotation angle in roll at 0°. After the extreme deviation comparison of bending moment (Fig 3), the most significant influence on the bending moment was the pedal backward intrusion (B), following with the pedal upward intrusion (C). The extreme deviation reaches 66.4 Nm concerning the influences of the pedal backward intrusion.

### 3.2 Ankle dorsiflexion angle

**Fig 4:**
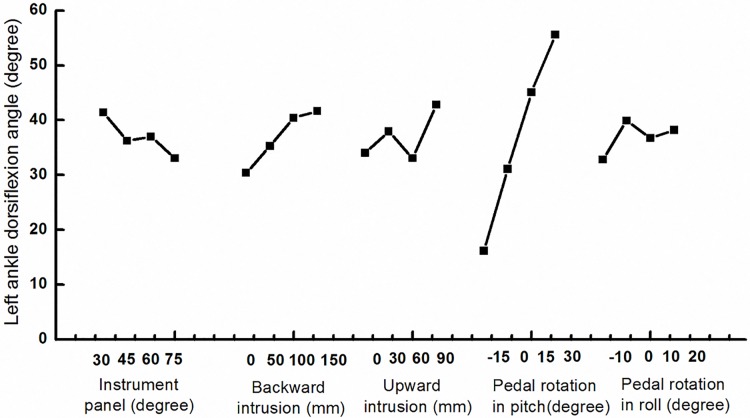
The influence trend of all the study parameters on left ankle dorsiflexion angle.

As shown in Fig 4, the most significant influence on ankle dorsiflexion angle was the change of the pedal rotation angle in pitch. The ankle dorsiflexion angle increased from 16.15° to 55.40°. While the pedal rotation angle in pitch results changed from -15° to 30° with the extreme deviation of 39.3 (Rd). The backward intrusion also showed that the same positive trend on the ankle dorsiflexion angle with a slight increase. With the increase of the IP angle the contact area would be larger, which would probably decrease the rotation angle. 9 out of all the 16 simulations recorded the failure of the tibia posterior cruciate ligament caused by the large ankle dorsiflexion angle. According to the tests, this failure would happen with the dorsiflexion angle at 38°-42°.

### 3.3 Combined ankle injury evaluation index

As the injury situation and Revised Tibia Index (RTI) index [21] shown in Table 2, it was noted that all RTI values were smaller than 1.0, which was considered as an injury threshold of the tibia. However, tibia fracture happened in a few cases. Lots of tibia fractures were located in ankle region. In this case, using RTI index to predict tibia fracture around ankle region cannot be reliable. In addition, previous studies also proved that the dorsiflexion angle can reflect ankle ligament injuries well [18]. Based on aforementioned ankle dorsiflexion influences, the maximum dorsiflexion angle was used together with RTI index to establish a robust evaluating index for ankle injuries including adjunctive tibia fractures. Thus, a combined ankle and tibia injury evaluation index (CAIEI) was suggested with coupling effects of dorsiflexion angle, axial force and bending moment. It was defined as Eq 1.

$$CAIEI=a_{1}\frac{A}{A_{c}}+a_{2}\left(\frac{F}{F_{c}}+\frac{M}{M_{c}}\right)$$ (1)

Wherein *a*_1_ and *a*_2_ were the weights for dorsiflexion angle, axial force and bending moment. *A*, *F* and *M* were the maximum dorsiflexion angle, the maximum tibia axial force measured near the ankle and maximum tibia bending moment measured near the ankle. *A*_*c*_, *F*_*c*_, *M*_*c*_ were the critical values of dorsiflexion angle, tibia axial force and bending moment regarding ankle injuries.

According to the previous studies, 36° was defined as the critical value for ankle dorsiflexion angle [18]. Critical values of 12 kN and 240 Nm from RTI index were selected for tibia axial force and bending moment thresholds, respectively [21]. Supposing ankle dorsiflexion angle and RTI index (including tibia axial force and bending moment) individually contribute 50% to ankle injury, the results for the CAIEI were shown in the Table 2. Most cases with ankle injury occurrences matched the CAIEI values more than 0.79 excepting for the case 8. Overall results also showed that larger CAIEI values represent more serious injury status with more injury occurrences.

## 4. Discussion

Overall occupant kinematics show that ankle injuries were largely related to the constraints from the IP, floor and pedal. When impact happens, the occupant would go forward related to cab due to the inertia. Occupants’ feet are most likely contact with the pedal and floor first. The consequent pedal intrusion and rotation probably will largely affect ankle injuries by resulting in excessive ankle rotation, bending moment and compression force. Meanwhile, hip with upper torso can go forward and can cause the contact between knee and IP. This contact force will pass through the tibia due to the inclining angle of IP, and significantly raise ankle injury risk by adding the compression force. So, five parameters including IP and pedal parameters were introduced to implement the parametric study for investigating ankle and tibia injuries during the frontal impact in the present study. As shown in Figs 3 and 4, proper IP angle can reduce injury related force, moment and human kinematics. Additionally, the usage of knee airbag can reduce tibia forward rotation, which will reduce tibia axial force and ankle rotation. Reducing pedal intrusion and rotation can significantly reduce ankle dorsiflexion, tibia force and tibia bending moment. All these can provide an approach to limit tibia and ankle injuries.

According to injury results, many tibia fractures are located around the ankle region. According to the RTI values shown in Table 2, some cases show none injuries with large RTI values more than 0.8 while others show serious injuries with RTI values lower than 0.7. The varying trend of RTI also can not reflect the variations of tibia injury severity. Although the RTI only reflects potential tibia injury risk, it also indicates that this index cannot demonstrate tibia fracture of all regions robustly as it is developed mainly by considering leg shaft injuries, especially for the tibia failure around ankle region. In addition, it is found that ligament failures in the ankle region happened in 5 out of 16 simulations in relation with the ankle rotation going over the range of physiological activities. Especially influences of ankle dorsiflexion on its ligament injuries are worthy of note.

The ankle dorsiflexion angle combined with RTI index is to establish a robust evaluating index for ankle injuries. The values of the new established index were shown in Table 2. The cases with large CAIEI values more than 0.98 generally represent multi ankle injury types. In the case 8, only ligament failure and tiny bone fracture showed a low CAIEI value. All none-ankle-injury cases showed a CAIEI value lower than 0.79. More complicated injury situation shows larger CAIEI values. The maximum CAIEI value appeared in the case 4, which present a complex multi-type injury with 6 regions. Other multi-type injury with more than 5 regions always showed a value close to or more than 1.0. So it can be concluded that larger CAIEI values is related to more complicated ankle injury situation in the present study. This indicates that the injury outcomes are in good correlation with the new index. However, the present index is mainly developed based on the simulation results. Thus, in the future the experimental study should be conducted to determine the weight values of the present index, in a further step to develop and validate the robustness of the present evaluation index.

## 5. Conclusion

The present study investigated occupant ankle and tibia injuries under realistic frontal impact environment using an integrated occupant-vehicle model, which was established by coupling an isolated car cab model and a hybrid occupant model with a biofidelic pelvis-lower limb model. A parametric study regarding intrusion and design parameters demonstrated the significant influences of the IP angle and pedal intrusion on tibia axial force, tibia bending moment and ankle dorsiflexion angle. As these three indexes were found to be significantly related to ankle and tibia injuries, a new evaluation index named CAIEI was established by coupling their influences to evaluate ankle and tibia injury risk and severity in robustness. Overall results indicated that the injury outcomes are in good correlation with this index, and also noted its necessity for future experimental studies.

## Supporting information

S1 Fig The integrated occupant-vehicle FE model and its validation. (TIF)

S2 Fig Evaluation of human body dynamic responses in simulation with experimental test results. (TIF)

S1 Table Summary of the verification of the FE lower extremity. (DOCX)

S2 Table Summary of material properties of lower extremities. (DOCX)
