# Supplementary figures and images for: Parametric analysis of occupant ankle and tibia injuries in frontal impact
Source: PLoS One. 2017 Sep 14;12(9):e0184521. doi: 10.1371/journal.pone.0184521 (PMC5598971; doi:10.1371/journal.pone.0184521)

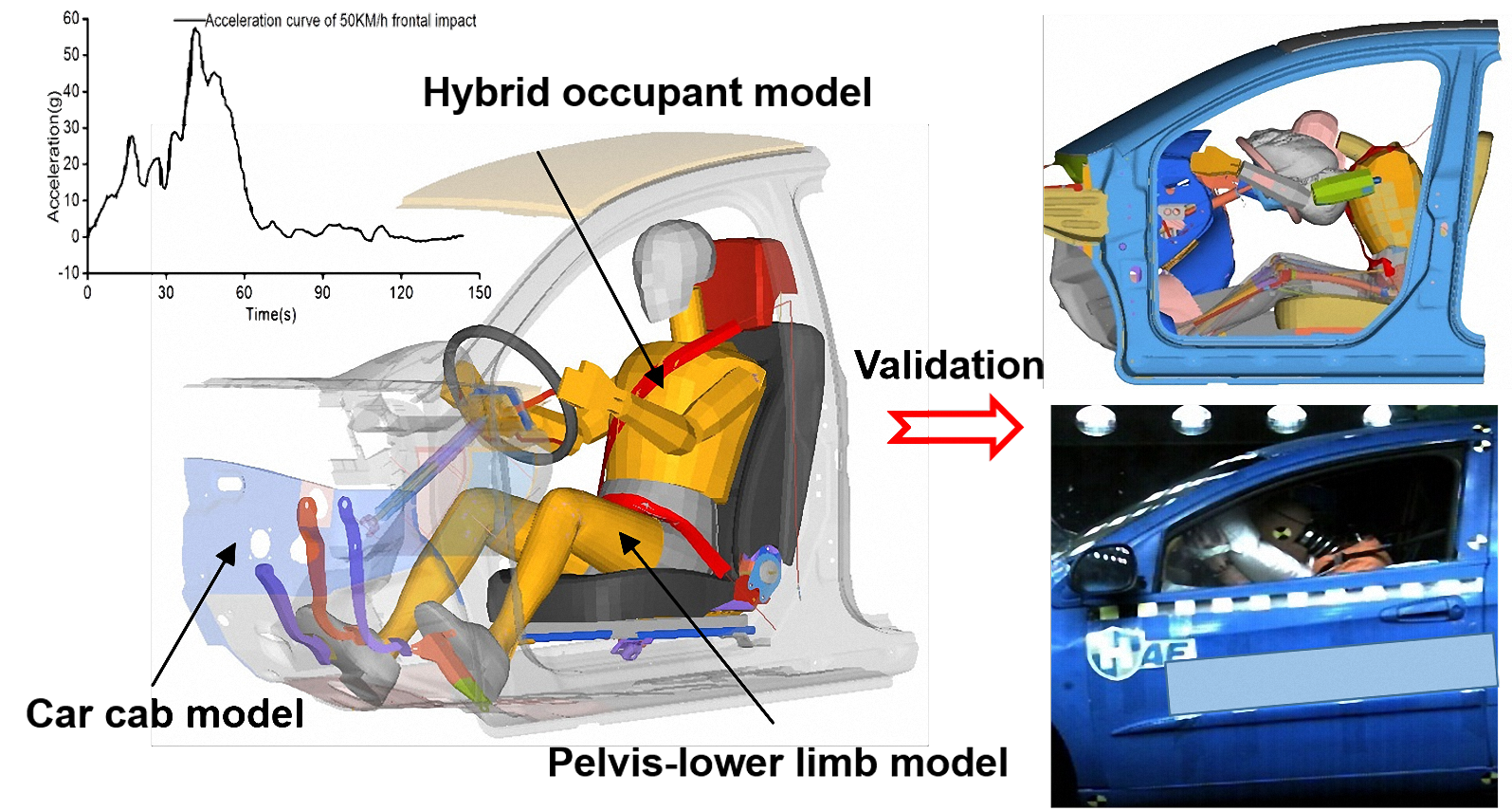

Supplement: S1 Fig — (TIF) [file pone.0184521.s001.tif]

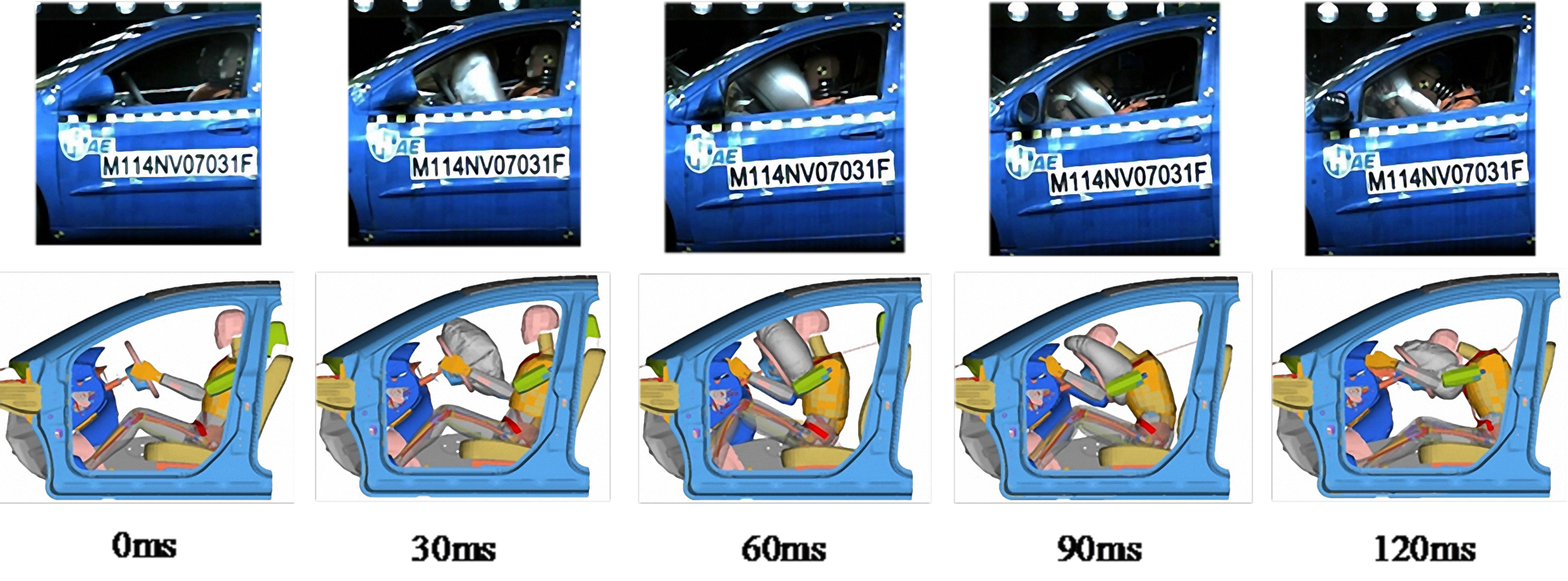

Supplement: S2 Fig — (TIF) [file pone.0184521.s002.tif]
