# Supplementary material for: Parametric analysis of occupant ankle and tibia injuries in frontal impact
Source: PLoS One. 2017 Sep 14;12(9):e0184521. doi: 10.1371/journal.pone.0184521 (PMC5598971; doi:10.1371/journal.pone.0184521)
Supplement: S1 Table — (DOCX) [file pone.0184521.s003.docx]

**S1 Table. Summary of the verification of the FE lower extremity**

|  | Experiment | Reference |
| --- | --- | --- |
| Component test | Quasi-static Femur three-point bending test | [1] |
|  | Dynamic Femur three-point bending test | [2] |
|  | Quasi-static Femur head static compression test | [3] |
|  | Dynamic PCL tensile test | [4] |
|  |  |  |
| Sub-system test | Dynamic Pelvis lateral impact test | [5] |
|  | Dynamic KT knee impact test | [6] |
|  | Dynamic Foot frontal impact test | [7] |
|  |  |  |
| Whole lower limb test | Dynamic Knee bolster impact test | [8] |
|  | Dynamic KTH knee impact test | [6] |

# References

1. Yamada H. Strength of Biological Materials. Springer, 1970.
2. Funk JR, Kerrigan JR, Crandall JR. Dynamic bending tolerance and elastic plastic material properties of the human femur. *The 48th annual proceedings association for the advancement of automotive medicine*, 2004, 48: 215–233.
3. Keyak JH, Rossi SA, Jones KA. Prediction of femoral fracture load using automated finite element modeling. *Journal of Biomechanics,* 1998, 31: p. 125-133.
4. Balasubramanian S, Beillas P, Belwadi A, Hardy WN. Below Knee Impact Responses using Cadaveric Specimens. *Stapp Car Crash Journal,* 2004, 48: p. 71-88.
5. Guillemot H, Besnault B, Robin S, Got C, Le Coz JY, Lavaste F, et al. Pelvic injuries in side impact collisions: a field accident analysis and dynamic tests on isolated pelvic bones, SAE Paper No: 973322, 1997.
6. Rupp JD, Reed MP, Chris AV, Kuppa S, Wang SC, Goulet JA, et al. The tolerance of the human hip to dynamic knee loading. *Stapp Car Crash Journal,* 2002. 46: 211-228.
7. Wheeler L, [Manning](http://xueshu.baidu.com/s?wd=author%3A%28Manning%2C%20P%29%20&tn=SE_baiduxueshu_c1gjeupa&ie=utf-8&sc_f_para=sc_hilight%3Dperson) P, Owen C, Roberts A, Lowne R, Wallace WA. Biofidelity of dummy legs for use in legislative car crash testing, 2000.
8. Hayashi S, Choi HY, Levine RS, Yang KH, King AI. Experimental and analytical study of knee fracture mechanisms in a frontal knee impact. SAE Paper No: 962423, 1996.
