# Supplementary material for: Parametric analysis of occupant ankle and tibia injuries in frontal impact
Source: PLoS One. 2017 Sep 14;12(9):e0184521. doi: 10.1371/journal.pone.0184521 (PMC5598971; doi:10.1371/journal.pone.0184521)
Supplement: S2 Table — (DOCX) [file pone.0184521.s004.docx]

**S2 Table. Summary of material properties of lower extremities**

| Material | Density  kg/m^3^ | Elasticity modulus  GPa | Poisson’s  ratio | Yield Stress  GPa | Failure of plastic strain | Reference |
| --- | --- | --- | --- | --- | --- | --- |
| Cortical bone | 2000 | 12-18.4 | 0.3 | 0.115-0.165 | 0.017-0.023 | [1-3] |
| Cancellous bone | 1100-1200 | 0.263-0.9 | 0.33 | 0.0037-0.0093 | 0.134 | [2, 4] |
| Muscle tendon | 1200 | 0.643 | 0.4 | 0.06 | 0.135 | [5] |
| Hip joint capsule | 1200 | 0.12 | 0.4 | 0.0061 | 0.08 | [6] |
| Cartilago articularis | 1800 | 0.045 | 0.40 | 0.003 | 0.2 | [7-8] |
| Meniscus | 1500 | 0.25 | 0.3 | － | － | [9] |
| Knee cruciate Ligament/accessory ligament | 1200 | 3.75(Bulk modulus) | c1=7.85; c2=0; c3=0.25; c4=60.4; c5=307.5 | | | [10-11] |

# References

1. Kemper A, Mcnally C, Kennedy E, Manoogian S, Duma S. The material properties of human tibia cortical bone in tension and compression: implications for the tibia index. *The 20th International Technical Conference on the Enhanced Safety of Vehicles Conference (ESV)*, Lyon, France, 2007.
2. Anderson AE, Peters CL, Benjamin DT, Weiss JA. Subject-Specific Finite Element Model of the Pelvis: Development, Validation and Sensitivity Studies. *Journal of Biomechanical Engineering,* 2005, 127: 364-373.
3. Nahum AM. Melvin, JW. Accidental Injury: Biomechanics and Prevention. Vol. Biomechanics of bone. New York: Springer-Verlag. 2002, 206–227.
4. Linde F, Hvid I, Pongsoipetch B. Energy absorptive properties of human trabecular bone specimens during axial compression. *Journal of Orthopaedic Research,* 1989, 7(3): 432-439.
5. Butler DL, Kay MD, Stouffer DC, Comparison of material properties in fascicle-bone units from human patellar tendon and knee ligaments. *Journal of Biomechanics,* 1986, 19(6): 425-432.
6. Hewitt J, Guilak F, Richard G, Vail TP. Regional material properties of the human hip joint capsule ligaments. *Journal of Orthopaedic Research,* 2001, 19(3): 359-364.
7. Repo RU, Finlay JB. Survival of articular cartilage after controlled impact. *The Journal of Bone and Joint Surgery,* 1977, 59-A (8): 1068-1076.
8. Dakin GJ, Arbelaez RA, Molz FJ, Alonso JE, Mann KA, Eberhardt AW. Elastic and viscoelastic properties of the human pubic symphysis joint: effects of lateral impact loading. *Journal of Biomechanical Engineering,* 2001, 123: 218-226
9. Fithian DC, Kelly MA, Mow VC. Material properties and structure- function relationships in the menisci. *Clinical Orthopaedics & Related Research,* 1990, 252: 19-31.
10. Untaroiu CD, Darvish K, Crandall J, Deng B, Jenne-Tai W. A finite element model of the lower extremity for simulating pedestrian impacts. *Stapp Car Crash Journal,* 2005, 49: 157-181.
11. Mo F, Arnoux PJ, Cesari D, Masson C. The failure modelling of knee ligaments in the finite element model. *International Journal of Crashworthiness.* 2012;17(6):630-636
